# The interaction effect of water deficit stress and seaweed extract on phytochemical characteristics and antioxidant activity of licorice (*Glycyrrhiza glabra* L.)

**DOI:** 10.3389/fpls.2024.1474399

**Published:** 2024-10-07

**Authors:** Vahid Fozi, Hassan Esmaeili, Abouzar Alizadeh, Ghasem Eghlima, Mohammad Hossein Mirjalili

**Affiliations:** ^1^ Department of Agriculture, Medicinal Plants and Drugs Research Institute, Shahid Beheshti University, Tehran, Iran; ^2^ Faculty of Agriculture, Lorestan University, Khorramabad, Lorestan, Iran

**Keywords:** antioxidant, phytochemical response, drought stress, licorice, seaweed extract

## Abstract

**Introduction:**

With increasing drought stress due to climate change and water scarcity, the agricultural sector has sought innovative strategies to mitigate the detrimental effects on crop productivity. One approach that has received significant attention is the use of fertilizers and biostimulants as potential means of alleviating drought stress.

**Methods:**

In this study, five different irrigation levels including 100% (control), 80% (slight stress), 60% (mild stress), 40% (moderate stress), and 20% (severe stress) of field capacity (FC) and seaweed extract (SWE) at three concentrations (0, 5, and 10 g/L) were applied to the pots containing one-year-old licorice (*Glycyrrhiza glabra L.*) plants in a factorial completely randomized design experiment with three replications for eight weeks.

**Results and discussion:**

The glycyrrhizic acid content increased with water stress intensity without the application of SWE until severe (20% FC) water stress treatment. The application of 10 g/L SWE under 100% FC led to a significant increase in the glycyrrhizic acid value (32.5±0.889 mg/g DW) compared with non-SWE application (30.0±1.040 mg/g DW). The maximum glabridin content (0.270±0.010 mg/g DW) was obtained under irrigation of 20% field capacity with 10 g/L SWE application. In addition, the activity of the all studied enzymes such as APX (ascorbate peroxidase), CAT (catalase), POD (peroxidase), and SOD (superoxide dismutase) were boosted by increasing the water stress levels. The use of SWE further enhanced the increase of some of these metabolites and enzymes, which, in turn, helped the plant to tolerate stress conditions through the scavenging of more ROS (Reactive oxygen species), wherein for this purpose, the SWE 10 g/L was more effective than other concentration. The plants efficiently eliminated ROS driven from drought stress by both non-enzymatic and enzymatic systems.

## 1 Introduction

Biostimulants are dose-dependent active ingredients that are classified in a group between plant growth regulators and fertilizers. Their benefits for plants include better absorption and assimilation of macro/micro elements, improvement in vegetative and reproductive growth, and the ability to withstand abiotic stresses (Franzoni et al., 2022). Various substances form the biostimulant complex, with the synergistic effect between the ingredients of the biostimulant leading to its effectiveness. One of the main classes of biostimulant based on the raw material source is algae extracts, which drive and stimulate some processes in plants, resulting in an improvement in the quantity and quality of the product. They also trigger the production of specialized (secondary) metabolites (SMs) in medicinal and aromatic plants (MAPs), which undeniably play a role in stress management.

Stress can cause an imbalance between the production of reactive oxygen species and their scavenging by intracellular production factors, leading to cell breakdown and, in severe cases, destruction (Najafi et al., 2021). According to the Intergovernmental Panel on Climate Change (IPCC) report, the Earth’s average temperature is increasing by 0.6° to 1° annually (Pant et al., 2021). One of the consequences of climate change is that plants are exposed to water stress and drought and uncultivable areas are increasing. Drought stress, one of the major abiotic stressors, forces MAPs to respond by producing SMs or radical-scavenging enzymes (Talbi et al., 2020). However, this stress reduces the growth and yield of and destroys their normal activities. MAPs can produce SMs in response to water deficit stress as one of their defense mechanisms. Studies have shown that specific transcription factors and regulatory genes play a mediating role in the response of MAPs to water deficit stress and the regulation of bioactive compound production (Hodaei et al., 2018; Qari and Tarbiyyah, 2021). As global climate change intensifies, the frequency, duration, and severity of drought events are expected to increase, posing substantial challenges to plant survival and agricultural sustainability (Tramblay et al., 2020). Plants respond to drought stress through a complex interplay of physiological, biochemical, and molecular mechanisms to conserve water, maintain cellular function, and ensure survival (Oguz et al., 2022).

Licorice (*Glycyrrhiza glabra* L.) is one of the important perennial MAPs of the legume family, whose raw materials are increasingly in demand in various industries due to its outstanding chemical compounds. Although licorice is known to be a tolerant plant to drought stress and is used to restore saline soils, the response to stress may vary depending on the plant genotype and developmental stage (Khaitov et al., 2021). Since many of this plant’s natural habitats in Iran have been destroyed by overharvesting, it is necessary to cultivate it due to the increasing demand for products derived from the plant (Esmaeili et al., 2020). On the other hand, many agricultural lands are facing water shortages due to successive droughts, and as farmers prefer to grow higher-yielding and annual crops, planning the cultivation of this valuable MAP requires scientific rehabilitation and utilization of poor and degraded pasture land.

Addressing the challenges posed by drought requires a focus on adaptation and resilience-building approaches in agricultural practice and policy. This includes promoting the use of drought-tolerant plant varieties, implementing water-efficient irrigation techniques, implementing agroforestry and soil conservation practices, and using biostimulants as a means of mitigating biotic and abiotic stress (Adhikari et al., 2019; Duygu, 2020; Abdoli and Ghassemi-Golezani, 2023; Bell et al., 2022).

Seaweed extracts (SWE) are natural biostimulants derived from various species of seaweed, such as brown algae (e.g., *Ascophyllum nodosum*, *Sargassum* spp., and *Ecklonia maxima*) and red algae (e.g., *Kappaphycus alvarezii*) (Ali et al., 2021). These extracts contain a diverse array of bioactive compounds, including plant growth regulators, amino acids, vitamins, and antioxidants, which can enhance plant tolerance to drought stress through multiple mechanisms (Khan et al., 2009). SWE promote the accumulation of osmolytes and osmoprotectants like proline, soluble sugars, and free amino acids in plant tissues (Ali et al., 2022). These compounds help maintain cellular turgor and membrane integrity and protect plants from dehydration under water-limited conditions (Chaves and Oliveira, 2004). SWE stimulate the synthesis of antioxidant enzymes and phenolic compounds, enhancing the plant’s ability to scavenge ROS and mitigate oxidative stress (Begum et al., 2021). The application of *Ascophyllum nodosum* extract in *Arabidopsis* upregulated genes involved in abscisic acid (ABA) signaling and antioxidant pathways, promoting drought tolerance (Santaniello et al., 2017). SWE represent a sustainable and effective approach to enhancing plant resilience to drought stress.

In previous studies, the response of licorice to water deficit has been investigated in different conditions (Xie et al., 2018; Hosseini et al., 2018; Khaitov et al., 2021; Haghighi et al., 2022; Zhang et al., 2023). However, no study has been conducted to uncover the effect of interaction between drought stress and seaweed extract on the phytochemical and biochemical characteristics of licorice. The results of this study can be exploited to grow this valuable medicinal plant in low-water areas using algae extract to prevent the harmful effects of drought on the plant.

## 2 Materials and methods

### 2.1 Chemicals and reagents

All the chemicals used, such as the reference standards of glabridin, glycyrrhizic acid, rutin, as well as Folin–Ciocalteu reagent, DPPH, and HPLC-graded solvents, were purchased from Sigma Aldrich Company. The soluble seaweed extract powder (*Ascophyllum nodosum* 90%) was prepared form Armansabz-Adine Company. The physical and chemical composition of the seaweed extract (SWE) employed in this study is presented in **Table 1**.

**Table 1:** Result of the seaweed extract (SWE) analysis derived from fresh *Ascophyllum nodosum* provided by the company.

Composite	Amount (%)
Total water-soluble nitrogen	2
Available phosphoric acid (P_2_O_5_)	3
Soluble potash	22
Amino-acid-free	8
Alginic acid	14.6
Mannitol	4

### 2.2 Plant materials and treatments

The seeds of Khoram Abad (Lorestan province) ecotype of licorice were collected for the current study. The seeds’ dormancy were broken using sulfuric acid according to a previous method (Haghighi et al., 2022); then, these were planted into suitable pots (12 L in volume) containing leaf mulch, sand, and a soil mixture with a ratio of 1:1:2 and transported to the greenhouse with a controlled atmosphere. Afterward, five different irrigation levels including 100% (control), 80% (slight stress), 60% (mild stress), 40% (moderate stress), and 20% (severe stress) of field capacity (FC) and SWE at three concentrations (0, 5, and 10 g/L) were applied to the pots containing 1-year-old plants in a factorial completely randomized design experiment with three replications containing three plants in each for 8 weeks (during May to July 2022). The irrigation cycle was determined for each treatment by measuring the weight of the pots and evaporated water and applying it every other day. The SWE was applied twice a week through foliar application. The result of the SWE analysis derived from fresh *Ascophyllum nodosum* provided by the company is presented in **Table 1**. At the end of the experiment, all the plants were harvested and underwent biochemical and phytochemical analyses.

### 2.3 Extraction and HPLC analysis

All plant materials including aerial parts and roots were dried at room temperature in a shaded place after 2 months of experiment. Furthermore, 500 mg of root sample was extracted with 50 mL ethanol/water (70:30) using ultrasonic (30 min) in three replications. The same amount of dried leaf was extracted by using 50 mL methanol using the same ultrasonic procedures. To assess the contents of glabridin and glycyrrhizic acid in the root samples and rutin in the aerial parts, 20 μL of all extracts was subjected to high-performance liquid chromatography, Waters 2695, in three replications. The separation process for the root and leaf compounds was performed in a C18 column (Knauer, 25 cm × 4.6 mm Eurospher 100-5) and Sunfire C18 column (Waters, 15 cm × 4.6 mm), respectively, as stationary phases. The mobile phase and gradient elution program were adjusted according to Esmaeili et al. (2019, 2020). A photodiode array detector was used to detect glycyrrhizic acid glabridin and rutin at 250, 230, and 200 nm, respectively. The standard solutions of glycyrrhizic acid, glabridin, and rutin were prepared at concentrations of 5, 10, 100, 200, and 1,000 ppm and were subjected to HPLC (Waters 2695) to obtain the calibration curves and following calculations.

### 2.4 Total phenolic and flavonoid content

Total phenolic content (TPC) was quantified according to Lister and Wilson (2001), wherein 25 µL of each extract was mixed with 125 µL Folin–Ciocalteu reagent 10% and 100 µL sodium carbonate solution 7.5% (V/V). After incubation in a dark place for 90 min, the absorbance of all wells was detected using a Biotek plate reader at 760 nm. A standard curve was constructed based on the different concentrations of gallic acid, and ultimately, the TPC was quantified and reported as milligrams of gallic acid equivalent per gram of dry plant material.

The total flavonoid content (TFC) was measured using an aluminum chloride (AlCl_3_) reagent (Zhishen et al., 1999). To quantification, 25 μL of each extract was added to a mixture containing 100 μL of distilled water and 7.5 μL of NaNO_2_ solution in three replications in a 96-well plate. At 6 min after the reaction initiation, 7.5 μL of AlCl_3_, 100 μL of NaOH, and 10 μL of distilled water were added to the each well. After 15 min, the absorption was detected by using a Biotek plate reader (EPOCH 2, USA) at the wavelength of 510 nm. Different concentrations of rutin were considered for plotting the calibration curve. The following equations *y* = 0.0003*x* + 0.7282 (*R* ^2^ = 0.989) and *y* = 0.0003*x* + 0.059 (*R* ^2^ = 0.991) were used to measure TPC and TFC, respectively.

### 2.5 DPPH, antioxidant capacity

According to Blois (1958), the free radical scavenging capacity of the methanolic extract of plant material was assessed using 2,2′-diphenypicrylhydrazyl (DPPH). After measuring the absorbance of the extract at 515 nm, the obtained values were utilized to calculate the radical scavenging capacity of the extracts using the following equation: inhibition (%) is calculated as (Ac − As)/Ac * 100, where As and Ac represent the sample and control absorbances, respectively. With the curve showing the relationship between inhibition percentage and extract concentration, the 50% inhibition concentration (IC_50_) of the extracts was assessed.

### 2.6 Antioxidant enzymatic assessment

Enzymatic activities were assessed using a spectrophotometric method. Initially, 500 mg of fresh leaves underwent homogenization with an extraction buffer consisting of potassium phosphate at pH 7. Subsequent centrifugation led to the isolation of the supernatant for the evaluation of catalase (CAT), peroxidase (POD), ascorbate peroxidase (APX), and superoxide dismutase (SOD) activities and determination of total protein content. The activity of catalase (CAT) enzyme was assessed by monitoring the reduction of hydrogen peroxide at a wavelength of 240 nm. A reaction mixture consisting of 50 µM phosphate buffer with a pH value of 7 and 15 µM hydrogen peroxide was prepared. The enzyme extract (50 µL) was added to reach a final volume of 1.5 mL and initiate the reaction. Changes in absorbance were recorded over a period of 60 s at a wavelength of 240 nm. Finally, enzyme activity was determined as absorbance changes per minute per fresh weight. POD activity assessment was conducted using guaiacol as a substrate at 470 nm. APX activity was evaluated by measuring the absorbance at 290 nm, wherein it showed the enzyme’s capability to oxidize the ascorbate per minute. SOD activity was quantified at 560 nm by determining the enzyme’s capacity to cause 50% reduction of nitroblue tetrazolium (NBT) as the reaction mixture consisted of 50 µM phosphate buffer, 12 µM methionine, 75 μM nitrobuterazolium (NBT), and 0.1 µM EDTA. To measure SOD activity, 50 μM of the enzyme extract and 10 μL of riboflavin were added to 1 mL of the mixture. The solution was then shaken and exposed to light from a fluorescent lamp for 15 min. Absorbance was recorded at a wavelength of 560 nm, and the results were expressed in milligrams of protein (Elavarthi and Martin, 2010).

### 2.7 Statistical analysis

All data were subjected to statistical analysis and two-way ANOVA (factor 1, drought stress; factor 2, SEW treatment) using R (version 4.3.3). Tukey’s test was used to identify the significantly different means (*P* < 0.05). All values are shown as mean ± standard deviation. R software was used to perform PCA and correlation analysis as well as to draw figures and graphs.

## 3 Results

### 3.1 Phytochemical traits

The result of ANOVA indicated that the effects of drought stress, SWE, and their interaction were significant on the glycyrrhizic acid and glabridin contents (**Supplementary Table S1**). Drought stress and SWE significantly altered the glycyrrhizic acid content compared with the control, either through their sole effects or their interaction. The glycyrrhizic acid content increased with water /stress intensity without the application of SWE until severe (20% FC) water stress treatment. The application of 10 g/L SWE under 100% FC led to a significant increase in the glycyrrhizic acid value (32.536 ± 0.889 mg/g DW) compared with non-SWE application (29.996 ± 1.040 mg/g DW). The same trend was also observed in the slight (80% FC) to moderate (40% FC) stress. The application of SWE in the severe (20% FC) water stress did not lead to a significant increase in glycyrrhizic acid content. The greatest glycyrrhizic acid content (44.440 ± 0.608 mg/g DW(was obtained in the plants treated with 40% FC and using 10 g/L SWE, while its minimum content (21.033 ± 1.000 mg/g DW) was observed in the plants exposed to 20% FC without SWE (**Table 2**).

**Table 2:** The effect of different levels of drought stress and seaweed extract (SWE) on phytochemical and antioxidant characteristics of licorice (*Glycyrrhiza glabra* L.).

Treatments		Phytochemical characteristics				
Irrigation levels (%FC)	Seaweed extract (g/L)	Glycyrrhizic acid (mg/g DW)	Glabridin (mg/g DW)	Total phenolic content (mg GAE/g DW)	Total flavonoid content (mg RE/g DW)	IC_50_ (μg/mL)
100	0	29.996 ± 1.040 h	0.123 ± 0.005 g	9.130 ± 0.241 g	11.626 ± 1.400 d	87.250 ± 1.800 a
100	5	30.346 ± 0.8420 gh	0.117 ± 0.005 g	9.136 ± 0.401 g	11.916 ± 1.560 d	83.430 ± 1.470 ab
100	10	32.536 ± 0.889 efg	0.137 ± 0.005 defg	10.640 ± 0.437 fg	12.190 ± 0.843 d	78.323 ± 0.965 bc
80	0	31.580 ± 1.020 fgh	0.130 ± 0.010 fg	10.790 ± 0.814 fg	12.180 ± 0.839 d	77.173 ± 1.820 c
80	5	32.866 ± 0.245 ef	0.133 ± 0.005 efg	10.853 ± 0.977 fg	12.366 ± 0.395 d	76.420 ± 1.040 cd
80	10	36.140 ± 0.650 cd	0.137 ± 0.015 defg	11.060 ± 0.727 f	12.393 ± 0.437 d	75.633 ± 2.620 cd
60	0	34.833 ± 0.297 de	0.157 ± 0.005 def	11.856 ± 0.376 ef	15.813 ± 0.409 c	73.813 ± 2.470 cde
60	5	37.310 ± 0.763 bc	0.160 ± 0.010 de	12.336 ± 0.633 ef	15.923 ± 0.650 c	71.760 ± 0.495 de
60	10	39.460 ± 1.250 b	0.163 ± 0.005 d	13.500 ± 0.538 de	16.340 ± 1.110 c	68.793 ± 1.190 e
40	0	37.966 ± 0.683 bc	0.210 ± 0.020 c	14.546 ± 0.451 cd	16.793 ± 0.595 c	61.970 ± 1.650 f
40	5	42.250 ± 0.455 a	0.220 ± 0.010 bc	14.810 ± 0.611 bcd	16.500 ± 0.531 c	59.546 ± 1.120 f
40	10	44.440 ± 0.608 a	0.237 ± 0.005 bc	15.473 ± 0.322 bc	18.476 ± 0.464 c	57.616 ± 2.700 fg
20	0	21.033 ± 1.000 i	0.240 ± 0.010 b	16.150 ± 0.238 bc	23.963 ± 1.440 b	53.920 ± 1.150 g
20	5	22.116 ± 0.645 i	0.243 ± 0.005 ab	16.510 ± 1.290 b	24.350 ± 1.330 b	52.456 ± 2.190 g
20	10	22.923 ± 0.696 i	0.270 ± 0.010 a	18.713 ± 0.434 a	28.066 ± 1.060 a	45.836 ± 2.190 h

Different letters in each column indicate significant differences (P < 0.01, Tukey’s mean difference test).

The glabridin content increased with the intensity of drought stress, wherein no significant difference was observed between the slight stress (80% FC) and the control (100% FC), but higher levels of stress led to a significant increase in the glabridin content. The maximum glabridin content (0.270 ± 0.010 mg/g DW) was obtained under irrigation of 20% field capacity with 10 g/L SWE application, while its minimum content (0.117 ± 0.005 mg/g DW) was recorded under 100% FC and 5 g/L SWE application. In the non-water stress (100% FC), the application of 10 g/L SWE increased the glabridin content (0.137 ± 0.005 mg/g DW), but its value was not significant compared with both 0 and 5 g/L SWE treatments (**Table 2**).

### 3.2 Total phenolic and total flavonoid contents

The result of ANOVA indicated that the effects of drought stress, SWE, and their interaction were significant on TPC and TFC (**Supplementary Table S1**). The root TPC significantly elevated with the intensification of drought stress compared with the control, except for the slight stress (80% FC). Irrigating the licorice plants with 20% FC and using 10 g/L SWE resulted in the highest c/ontent of root TPC (18.713 ± 0.434 mg GAE/g DW), while the lowest TPC (9.130 ± 0.241 mg GAE/g DW) was observed in the plants subjected to 100% field capacity (FC) without using SWE (**Table 2**).

The TFC was significantly influenced by drought stress under mild to severe water stress, wherein no significant difference was recorded for TFC between 100% and 80% FC. The application of 10 g/L SWE led to a significant increase in TFC only under severe (20% FC) drought stress, indicating the highest TFC (28.066 ± 1.060 mg RE/g DW) (**Table 2**).

### 3.3 Total antioxidant

The result of ANOVA showed that the total antioxidant activity was significantly influenced by drought, SWE, and their interaction (**Supplementary Table S1**). In this study, different levels of irrigation and SWE application significantly influenced the antioxidant capacity of the extracts as measured by IC50 values. However, among all treatments, the lowest IC_50_ value (45.836 ± 2.190 μg/mL) was obtained under irrigation of 20% field capacity (FC) and using 10 g/L SWE. Conversely, the highest IC_50_ value (87.250 ± 1.800 μg/mL) was observed under 100% FC without the use of SWE. A positive correlation was observed between the radical scavenging ability of extracts and the intensity of drought stress (**Table 2**).

### 3.4 Antioxidant enzyme activity

The result of ANOVA revealed that both drought stress and SWE as well as their interaction significantly influenced the enzymatic activity of extracts based on the quantification of APX (ascorbate peroxidase), CAT (catalase), POD (peroxidase), and SOD (superoxide dismutase) (**Supplementary Table S1**).

**Figure 1:**
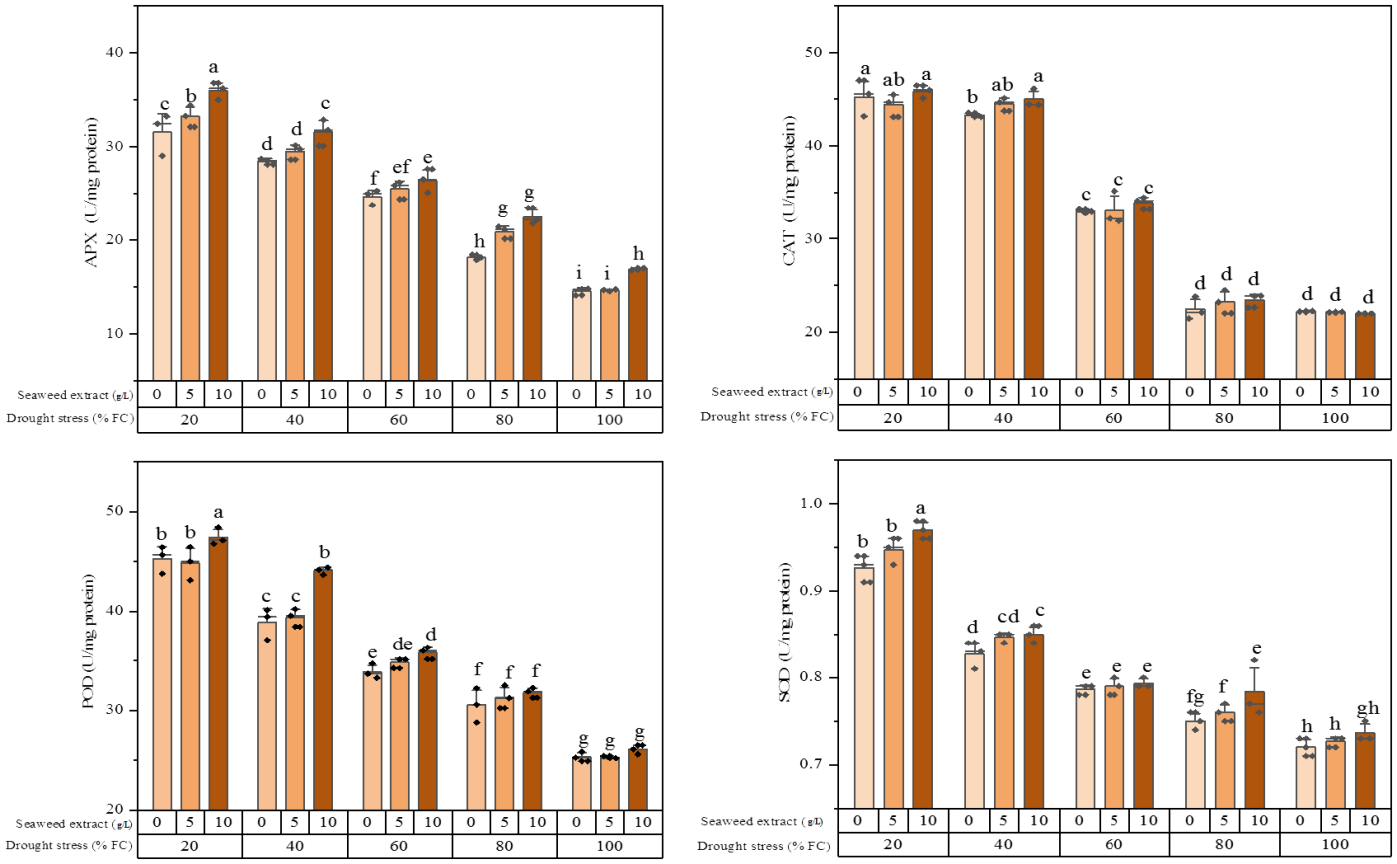
Antioxidant enzymes (CAT, POD, APX, and SOD) activity of the licorice (*Glycyrrhiza glabra* L.) plant under drought stress and seaweed extract (SWE) treatments.

It was found that the activity of all the studied enzymes was boosted by increasing the water stress levels (**Figure 1**). The plants exposed to 20% FC combined with 10 g/L SWE application exhibited the highest APX activity (36.0 ± 0.92 U/mg protein), which showed a significant difference with application of 5 g/L and 0 g/L SWE. The plants subjected to 100% FC and without SWE application showed the lowest APX activity (14.6 ± 0.38 U/mg protein). In the non-water stress condition, the plants treated with 5 g/L SWE did not cause a significant change in the activity level of the APX enzyme, while the effect of 10 g/L SWE was significant.

The results of the CAT enzyme activity analysis showed that there was no significant difference between 100% FC and slight (80% FC) stress conditions. There was also no significant difference in the activity of this enzyme between moderate (40% FC) and severe (20% FC) stresses with 5 g/L and 10 g/L SWE application. The highest CAT activity (45.9 ± 0.69 U/mg protein) was recorded in the plants exposed to 20% FC and using 10 g/L SWE. Conversely, the minimum CAT activity (2/2.0 ± 0.02 U/mg protein) was recorded in the plants treated with 10 g/L SWE without water deficit stress.

An increment in the POD activity was observed with an increase in the level of water stress, as the highest enzyme activity was related to 20% FC. The use of SWE under 100% FC and 80% FC irrigation regimes did not change the POD activity, while utilizing 10 g/L SWE under 40% FC and 20% FC led to a higher POD enzyme activity. The minimum POD activity (25.3 ± 0.12 U/mg protein) was observed under full irrigation and 5 g/L SWE application.

The SOD activity showed an almost similar trend with the POD enzyme, wherein the plants subjected to 20% water stress and using 10 g/L SWE exhibited the highest SOD activity (0.97 ± 0.01 U/mg protein). Conversely, the minimum SOD activity (0.72 ± 0.01 U/mg protein) was observed with irrigation of 100% FC without SWE application.

### 3.5 Proline content

**Figure 2:**
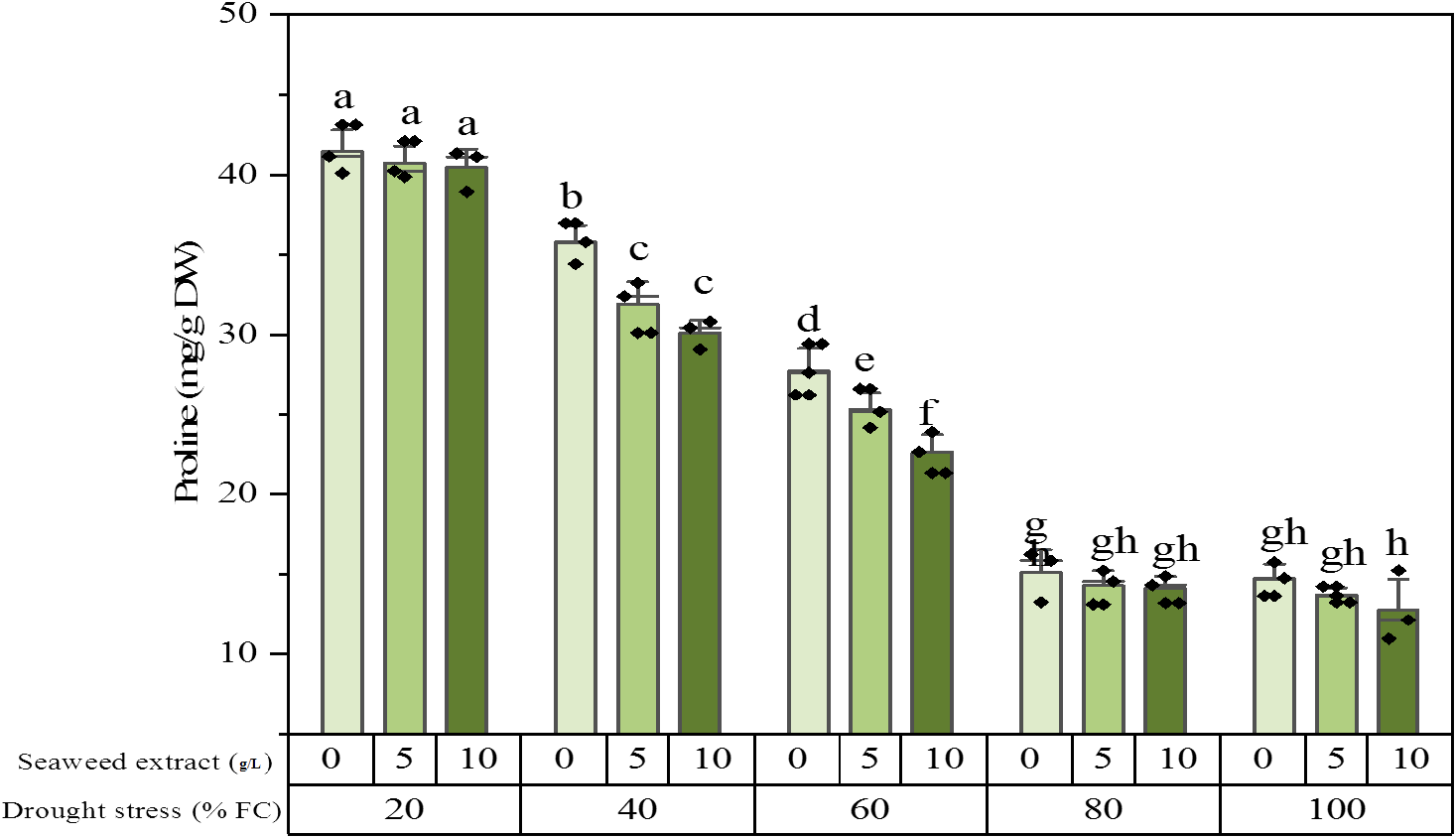
Proline content of the licorice (*Glycyrrhiza glabra* L.) plant under drought stress and seaweed extract (SWE) treatments.

**Figure 3:**
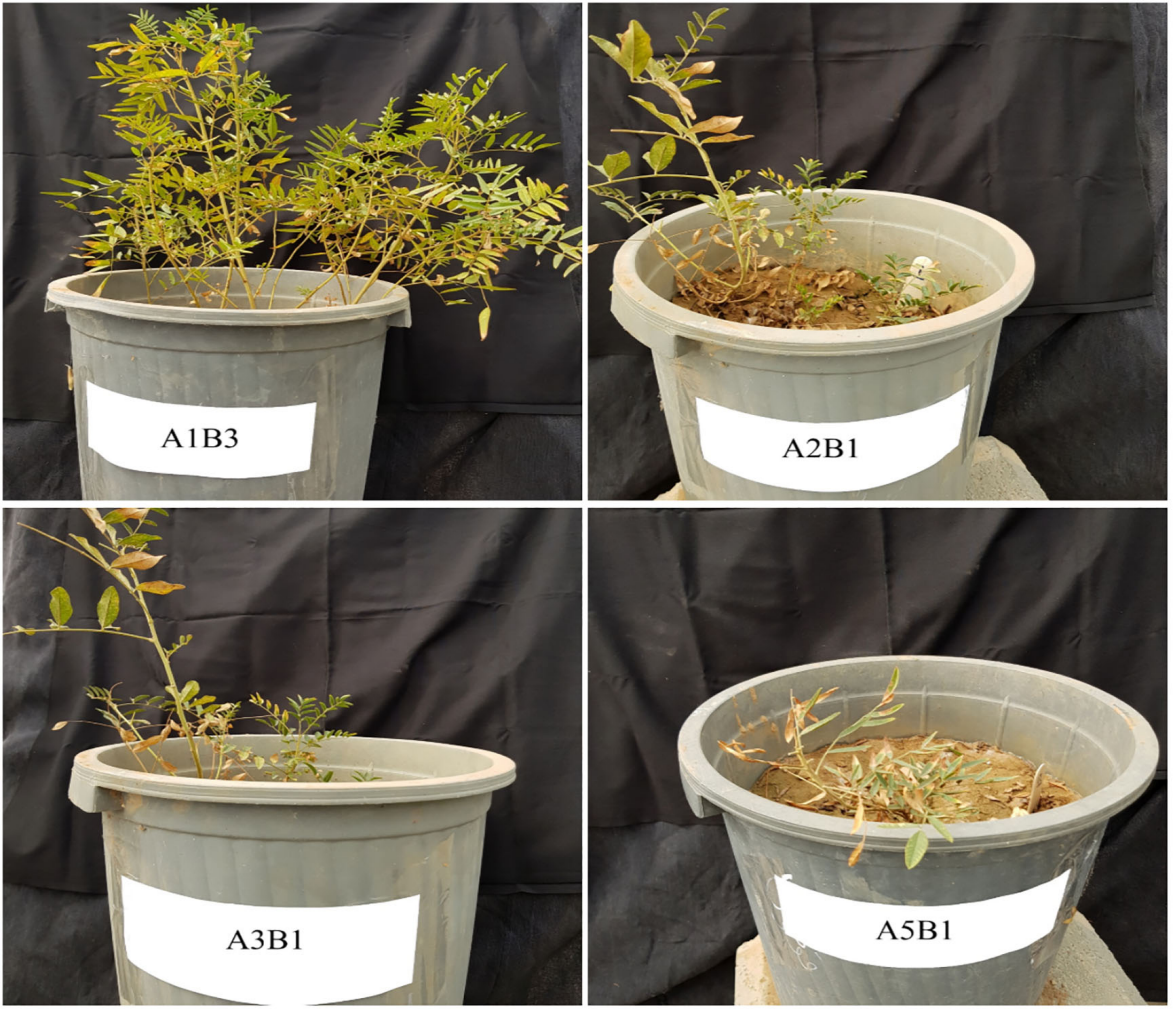
Growth status of licorice in some treatments (*Glycyrrhiza glabra* L.). A1B3: 100% FC irrigation and SWE 10 g/L. A2B1: 80% FC irrigation without SWE. A3B1: 60% FC irrigation without SWE. A5B1: 20% FC irrigation without SWE.

The result of ANOVA showed that drought stress, SWE, and their interaction significantly altered the proline content of the plant extracts. The proline content reached its maximum value equal to 41.5 ± 1.54 (mg/g DW) under 20% FC irrigation without SWE, while it dropped to 12.8 ± 2.20 (mg/g DW) under 100% FC irrigation and application of SWE 10 g/L. In the plants exposed to slight stress (80% FC) and full irrigation (100% FC), there was no difference in the proline level. In the mild (60% FC) and moderate (40% FC) stresses, the proline content was significantly decreased by SWE utilization (**Figure 2**). The growth status of the plants in some treatments is shown in **Figure 3**.

### 3.6 Correlation among traits

**Figure 4:**
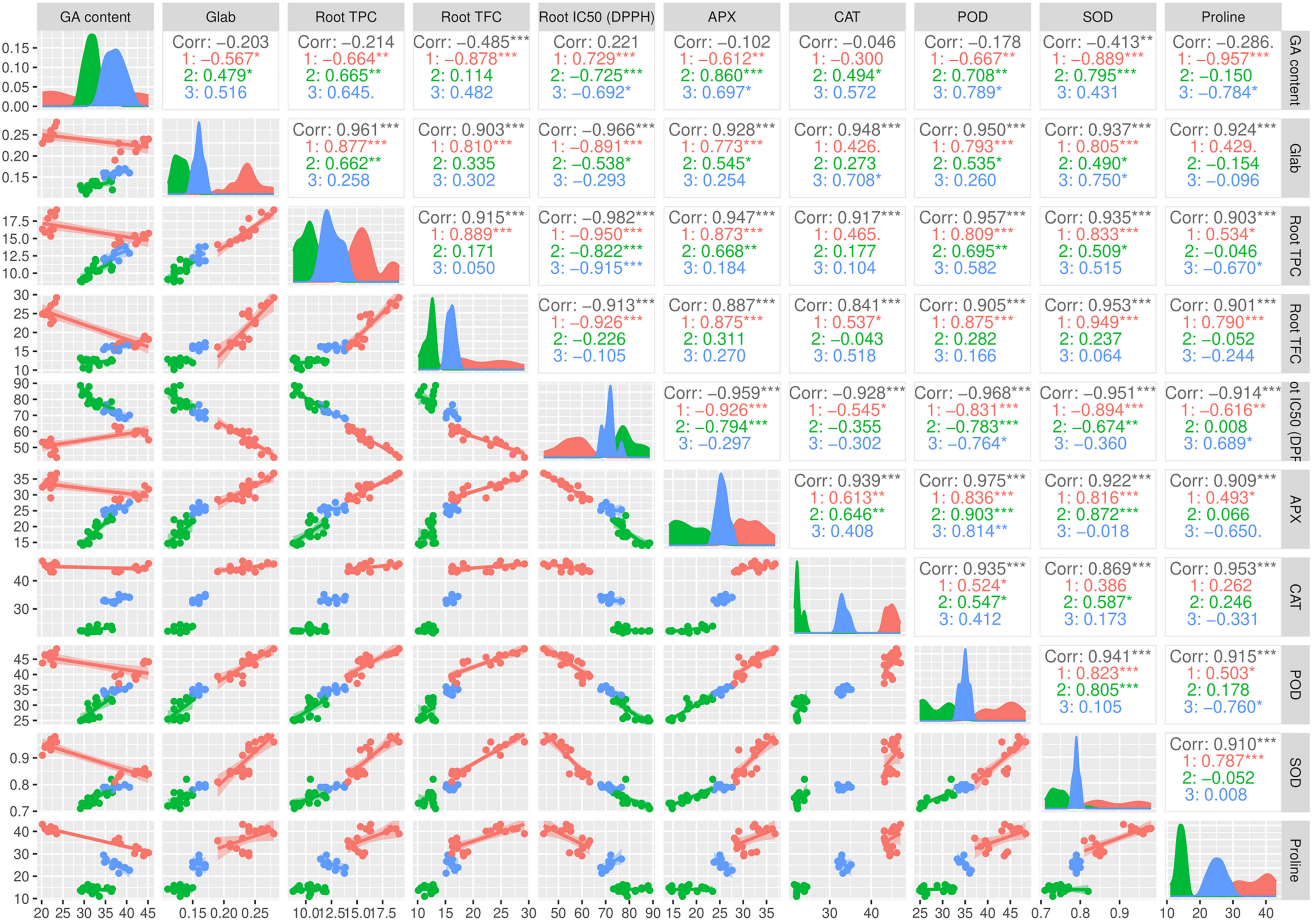
Pairwise matrix, density plots, and scatter plots among the studied traits of the licorice (*Glycyrrhiza glabra* L.) plant under drought stress and seaweed extract (SWE) treatments. *, **, ***, indicate significant levels at 0.05, 0.01, and 0.1, respectively.

**Figure 5:**
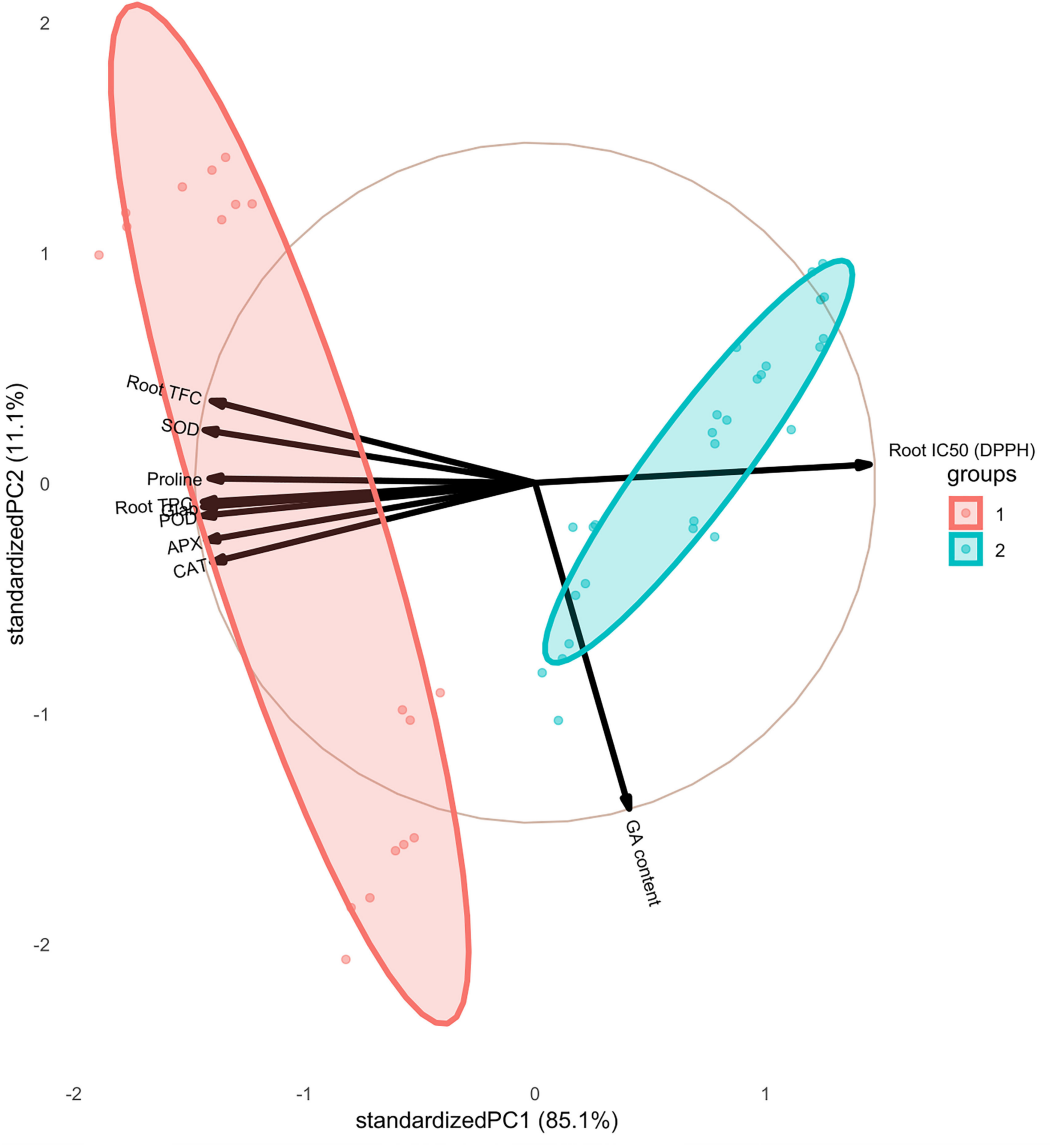
Biplot diagram based on PC1 and PC2 among the studied traits of the licorice (*Glycyrrhiza glabra* L.) plant under drought stress and seaweed extract (SWE) treatments.

The pairwise matrix, density plots, and scatter plots among the studied traits are presented in **Figure 4**. The correlation between the traits measured in this experiment showed that glabridin content showed a strong and significant positive correlation with all the antioxidant enzymes, root TPC, and root TFC. The content of glabridin also had a significant negative relationship with IC_50_. In previous studies, the antioxidant role of glabridin has been mentioned as an important component of licorice root (Esmaeili et al., 2019). The negative correlation between total phenol and flavonoid content with IC_50_ was confirmed in this test, as expected. The glycyrrhizic acid (GA) content also had an inverse relationship with the measured antioxidant enzymes, which was not significant except in the case of SOD. The biplot diagram was drawn as a powerful tool to visualize the PCA results, which help to understand the internal structure of the data and the relationship between variables and samples (**Figure 5**). The biplot diagram based on PC1 and PC2 confirmed the results of the correlation analysis, wherein the total phenol and flavonoid contents and antioxidant enzymes were placed in one group and direction and the IC_50_ content was placed on the opposite side.

## 4 Discussion

### 4.1 Interaction effect of drought and SWE on phytochemical traits

The biological and pharmaceuticals properties of *G. glabra* have been firstly attributed to two of its valuable compounds, triterpenoid saponins and flavonoids (Li et al., 2010). *Glycyrrhizinic acid* as a triterpenoid saponin and glabridin as a flavonoid are two valuable and biologically active compounds with extensive applications. Glycyrrhizin is an oleanane-type triterpenoid saponin found in the amount of 2% to 8% in the roots and stolons of *Glycyrrhiza* species, and the demand for its consumption in medicine, industry, cosmetics, health, and food is increasing day by day (Esmaeili et al., 2019). Glabridin, a prenylated isoflavan is another valuable phytochemical compound isolated from the plant’s underground parts which have received considerable attention in recent years. Glabridin is the most important flavonoid in licorice and is considered a phytoestrogen compound (Esmaeili et al., 2024). The SM content usually elevated during drought stress, which depends on the plant species and the intensity and duration of the stress period (Mahajan et al., 2020). In this regard, drought stress can effectively act as a controlling factor to increase the yield of compounds like alkaloids, phenols, and isoprenoids (Shil and Dewanjee, 2022). In a study, Khaitov et al. (2021) demonstrated that licorice roots produced the highest glycyrrhizic acid content in response to low or moderate water deficit, enhancing the quality of the raw materials, while this metabolite significantly decreased in the severe water stress condition. On the other hand, the findings of Nasrollahi et al. (2014) showed that extreme drought stress elevated the glycyrrhizic acid content in the *G. glabra* stolons through the expression of β-amyrin synthase (bAS) and sequalene synthase (SQS) genes. Sarker and Oba (2018) have demonstrated that the SM contents of *Amaranthus tricolor* plant were highly dependent on drought stress, wherein under control conditions the concentrations of all the flavonoids and phenolic acids were dramatically lower than those in the severe drought conditions. They suggested that the cultivation of this plant in arid and semi-arid areas can lead to the production of more bioactive compounds and thus a higher nutritional value for the human diet. In this study, the main therapeutic metabolite content of licorice, glycyrrhizic acid, significantly increased with the intensification of drought stress levels compared with the control (100% FC), except for the severe water stress (20% FC), while the glabridin content was positively correlated with an increase in stress level. The SMs such as glycyrrhizic acid and glabridin are known to play a significant role in alleviating abiotic stress, as a non-enzymatic plant defense system against stress (Mousavi et al., 2022). Abbasi and Mohammadi (2023) reported that moderate to intense drought stress can positively affect the accumulation of glycyrrhizic acid and glabridin in *G. glabra*. According to Yao et al. (2022), under drought stress, licorice underground parts exhibited a significant accumulation of glabridin and glycyrrhizic acid contents. A transcriptome analysis of drought-treated licorice revealed a significant enrichment of upregulated differentially expressed genes (UDEGs) involved in the synthesis of flavonoids in both the aerial and underground parts of the plant and glycyrrhizic acid in the latter. Furthermore, under drought stress, the accumulated JA content was found in both underground and aerial parts. Additionally, they reported that through the JA-mediated signaling pathway, drought stress may facilitate the accumulation of active ingredients in pharmaceuticals.

Although changes in plant metabolites in response to drought stress have been widely discussed, the interaction between drought stress and SWE has rarely been investigated. Our result indicated that under optimal water conditions (100% FC), the application of SWE 10 g/L resulted in a significant increase in glycyrrhizic acid content, while this effect was not significant in the case of glabridin. This aligns with the findings of Elansary et al. (2016) and Stirk et al. (2020) which indicate that the application of SWE on unstressed plants can stimulate the biosynthesis of saponins and isoflavones.

In this experiment, an increase in the production of glycyrrhizic acid when using SWE under slight to moderate water deficit stress is probably due to the role of this biostimulant in moderating the stress conditions for the plant. Our findings further revealed that only the application of SWE 10 g/L effectively raised the glabridin content in the severe drought stress condition. Glycyrrhizic acid and glabridin have been recognized as stress-dependent metabolites (Yao et al., 2022).

SWEs have been shown to increase plants’ resistance to both biotic and abiotic stress in numerous instances. More understanding of the mechanisms of action has been made possible by recent developments in the use of molecular tools. Evidence suggested that SWEs also contain molecules that stimulate plant metabolism and defense responses by up- and downregulating genes involved in the biosynthesis of hormones, even though some responses are attributed to different physiologically active compounds present in the complex mixture that alter physiological processes (Wally et al., 2013). Plants show different reactions to SWE depending on the method of application and dosage (Spann and Little, 2011). SWE treatment may influence the nutritional composition of vegetable crops by raising phenolics, flavonoids, and antioxidant capacity (Fan et al., 2011). According to a study on *Calibrachoa x hybrida*, SWE considerably raised the amount of tannin, flavonols, and total phenolic content in plant leaves, which, in turn, raised the antioxidant activity of leaf extracts (Elansary et al., 2016). Pelargonium cuttings treated with SWE showed increases in their leaves’ phenolic content (Krajnc et al., 2012), while in another study the effect of the extract on the increase of phenol and flavonoids was not observed (Xu and Leskovar, 2015). According to recent research, antioxidant enzymes and secondary metabolic pathways, particularly the biosynthesis of flavonoids (Santaniello et al., 2017), are activated during SWE treatments, which help plants adapt to stressful conditions.

### 4.2 Interaction effect of drought and SWE on TPC, TFC, and total antioxidant

Plant SMs, including phenolic and flavonoids compounds, which harbor antioxidant capacities, serve as primary molecules by which plants respond to abiotic stresses (Jan et al., 2021). Drought stress can alter the biosynthesis pathways of terpenoids and shikimate, leading to upregulation of isoprenoids, polyketides, and polyphenols and thus their increased accumulation (Park et al., 2023; Tahat and Al-Momany, 2021). Plants rely on SMs, such as phenols and flavonoids, to withstand drought conditions. Castellarin et al. (2007) reported a significant increase in flavonoids and phenolic compounds in grape and pea plants under drought stress. In the present study, mild to severe drought stress caused a significant increase in the accumulation of phenolics and flavonoids. The application of SWE 10 g/L on the plant under severe drought stress was associated with a remarkable increase in TPC and TFC. SWE has been extensively utilized to alleviate the damage caused by drought stress, which is primarily due to oxidative stress (Mansori et al., 2015). *Ascophyllum nodosum* extract significantly increased the amount of phenolics and flavonoids in two different broccoli cultivars under no stress condition (Lola-Luz et al., 2014). The nutrient profile of SWE, including macro-elements, microelements, vitamins, auxins, and phytohormones, affects plant growth and metabolism by transporting osmolytes, regulating membrane permeability, and promoting the production of stress-alleviating amino acids. These adjustments help regulate water capacity in plants (Khan et al., 2022).

The potential antioxidant activity of the licorice methanolic extracts was assessed using the DPPH method. The IC_50_ value represents the concentration of the extract required to scavenge 50% of the DPPH radicals. A lower IC_50_ value signifies a higher antioxidant potency. Esmaeili et al. (2019) reported an average IC_50_ value of 73.7 (μg/mL) for various populations of *G. glabra* collected from their natural habitats. In a similar study, Rebey et al. (2012) investigated the free radical scavenging capacity of *Cuminum cyminum* extract under drought conditions and demonstrated a significant increase in the DPPH scavenging activity by 17.40% and 64.05% under mild and severe water deficit stress, respectively, which is consistent with our results. They also reported a significant positive correlation (*r* = 0.92 at *p* < 0.05) between phenolic content and the capacity to scavenge the DPPH free radicals under drought conditions. This aligns with our findings, as the extract with the highest phenolics content possessed the highest antioxidant capacity. In this study, under non-drought stress condition, the lowest IC_50_ value was observed when utilizing SWE 10 g/L. Nikoogoftar-Sedghi et al. (2023) performed a similar study on foliar application of *Ascophyllum nodosum* extract in pistachio and reported that SWE was able to enhance the radical scavenging capacity by 22.53% under drought stress. This is also consistent with the study of Kumari et al. (2021), which demonstrated that, in a stress-free environment, SWE treatment increased the DPPH radical scavenging activity in *Vigna radiata*. Similarly, in our study, the antioxidant capacity increased with both intensifying drought stress and application of SWE. The phenolics and antioxidants in seaweed extract might alter the primary and secondary metabolic pathways in the treated plants, ultimately leading to improved tolerance toward abiotic stresses (Ammar et al., 2017; Kasim et al., 2016; Khan et al., 2009).

### 4.3 Interaction effect of drought and SWE on antioxidant enzyme activity

In plant cells, the ascorbate–glutathione cycle serves as a powerful hydrogen peroxide detoxifying system under abiotic stresses. In this cycle, ascorbate peroxidase (APX) isoenzymes catalyze the conversion of H_2_O_2_ into H_2_O, utilizing ascorbate as a specific electron donor in the chloroplast (Correa-Aragunde et al., 2013; Smirnoff, 2018; Wani et al., 2021). Within the context of cellular redox homeostasis and redox signaling, two non-enzymatic antioxidants play crucial roles: ascorbate (AsA) and glutathione (GSH). These antioxidants are regulated by genes encoding APXs, which directly or indirectly contribute to enhancing the photosynthetic rates in plants under adverse environmental conditions (de Pinto et al., 2013; Hasanuzzaman et al., 2019). There was an upregulation in the expression of the APX-coding gene in *Phaseolus vulgaris* under drought stress, indicating a key role of molecular regulation mechanisms induced by abiotic stresses (Nageshbabu and Jyothi, 2013). There was a significant increase in the activity of APX in soybeans under drought stress (Kausar et al., 2012). In the present study, the accumulation of APX enzyme significantly increased in the drought-stressed plants when treated with 10% SWE. Elansary et al. (2017) investigated the alleviation effect of *Ascophyllum nodosum* extract in a prolonged irrigation treatment in Salam Turfgrass, wherein the activity of APX(s) was significantly higher compared with the plant exposed to SWE in both stress and normal condition. The increase in APX activity following the use of seaweed extract contributed to enhancing drought stress tolerance by providing antioxidants (Frioni et al., 2018).

Drought-stressed plants maintain CAT activity in their leaves to scavenge the photorespiratory H_2_O_2_ produced as a result of drought stress, especially under severe water deficit stress (de Pinto et al., 2013). Plants, to a certain extent, are efficiently able to eliminate ROS by both non-enzymatic and enzymatic antioxidants under stress conditions. However, the oxidative stress will occur when the quantity of produced ROS surpasses the plant’s antioxidant system (Ajithkumar and Panneerselvam, 2014; Vanderauwera et al., 2012). It has been reported that CAT is an enzyme that reduces the damage caused by drought and salt stress through scavenging the ROS and preventing oxidative stress (Mittler et al., 2011; Sachdev et al., 2021). Our results showed that CAT activity and its accumulation are positively associated with the severity of stress, which is consistent with some other studies on drought stress (Faize et al., 2011; Mittler et al., 2011; Pinheiro and Chaves, 2011). Hosseini et al. (2018) reported a 1.6- to 3.7-fold increase in CAT activity in *G. glabra* plants under drought stress, which is consistent with our data showing about a twofold increase in CAT activity under stress conditions without SWE application. In the present study, CAT activity did not show a significant difference when drought-stressed plants were treated with SWE. Contrary to the results of this study, Hernández-Herrera et al. (2024), who conducted a study on the amelioration effect of another brown algae (Phaeophyceae) extract on tomato plants under salinity stress, reported that CAT activity nearly doubled under saline irrigation, while it decreased by approximately 6.63% with the application of seaweed extract.

The H_2_O_2_ released under abiotic stresses such as water deficiency on *Glycyrrhiza uralensis* is scavenged by peroxidase (POD), which reduces H_2_O_2_ using glutathione (GSH) and glutathione reductase (GR) (Zhang et al., 2017). Peroxidase, an iron heme protein, plays a key role in accelerating the reduction of H_2_O_2_ in the cell wall (Özdemir et al., 2004). POD is also an important enzyme in scavenging H_2_O_2_ produced in plants’ chloroplasts under abiotic stress (Dumanović et al., 2021). There was a significant increase in POX activity in three chickpea (*Cicer arietinum*) cultivars under drought stress (Mafakheri et al., 2011). Reducing the toxic levels of H_2_O_2_ during cell metabolism is entirely dependent on POX activity, representing an adaptation that effectively protects cells against oxidative damage (Ahmad et al., 2010). In the present study, drought stress significantly altered POD activity, resulting in a 1.53-fold increase in plants irrigated with 40% FC compared with the control. Application of SWE 10 g/L on stressed plants helped alleviate oxidative stress, with a significant increase observed in the POD levels under mild to severe stress. Interestingly, the higher concentrations of SWE were more effective in increasing the POD levels, which directly correlates with the maintenance of cellular homeostasis and scavenging of ROS. Similar results were reported by Nikoogoftar-Sedghi et al. (2023) in a study that investigated the effect of foliar application of *Ascophyllum nodosum* extract on pistachio in a stress condition.

In the present study, the plants subjected to 20% water stress and using 10 g/L SWE exhibited the highest SOD activity (0.97 ± 0.01 U/mg protein). A 1.7- to 4.9-fold increase in SOD accumulation in different wild populations of *G. glabra* under drought stress (Hosseini et al., 2018) has been reported. The primary scavenger of free radicals in plants is SOD, which produces H_2_O_2_. Then, CAT and POD remove the H_2_O_2_, leading to a homeostatic balance that contributes to plant survival (Golashan et al., 2011; Khatun et al., 2008). In a transgenic *Arabidopsis*, the enzymatic activity of superoxide dismutase and catalase was increased, resulting in reduced damage caused by H_2_O_2_ (Huang et al., 2015). There was a significant difference in SOD activity in drought-stressed plants compared with control plants (Mansori et al., 2015).

### 4.4 Interaction effect of drought and SWE on proline content

Drought stress is usually associated with proline accumulation in licorice (Haghighi et al., 2022). Hosseini et al. (2018) reported that intense drought stress was able to increase the proline concentration in the roots and leaves of licorice by 2.5 to 4.5 times, respectively. The increase in proline levels in cells is one of the important key factors in many plant species to reduce the oxidative effects of drought stress (Moreno-Galván et al., 2020). Healthy cell growth and effective photosynthesis are highly dependent on the presence of osmotic substances, which are responsible for adjusting the osmotic pressure in leaf cells and reducing water potential (Lin et al., 2024). However, applying SWE to the plants resulted in a decrease in the level of proline, indicating a reduction in stress levels. As an antioxidant, proline contributes to inhibiting lipid peroxidation and scavenging free radicals (Zulfiqar and Ashraf, 2023). In this study, plants accumulated a higher content of proline as the level of drought stress increased. Our results were aligned with the results of Hernández-Herrera et al. (2024), which reported that drought stress increased the proline content as a stress indicator. However, the utilization of seaweed extract was able to decrease the level of proline, alleviating the stress condition.

## 5 Conclusion

In this study, the phytochemical and biochemical response, respectively, of licorice plant to drought stress and seaweed extract (SWE) application were investigated. As the intensity of drought levels increased, the plant tried to overcome the stress conditions and maintain the homeostasis state of the cells by increasing the production of some metabolites like glycyrrhizic acid and glabridin and antioxidant enzymes such as APX, CAT, POD, and SOD. The use of SWE further enhanced the increase of some of these metabolites and enzymes, which, in turn, helped the plant to tolerate stress conditions through the scavenging of more ROS, wherein, for this purpose, SWE 10 g/L was more effective than the other concentrations. The results of this study can be exploited to grow this valuable medicinal plant in low-water areas using algae extract to prevent the harmful effects of drought on the plant.

## Data Availability

The original contributions presented in the study are included in the article/**Supplementary Material**. Further inquiries can be directed to the corresponding author.
