# Supplementary material for: The interaction effect of water deficit stress and seaweed extract on phytochemical characteristics and antioxidant activity of licorice (Glycyrrhiza glabra L.)
Source: Front Plant Sci. 2024 Oct 7;15:1474399. doi: 10.3389/fpls.2024.1474399 (PMC11491410; doi:10.3389/fpls.2024.1474399)
Supplement: Supplementary file 1 [file Table1.docx]

| **Table S1**. The analysis of variance (ANOVA) for phytochemical traits of *Glycyrrhiza glabra* affected by drought stress and seaweed extract | | | | | | |
| --- | --- | --- | --- | --- | --- | --- |
| Sources of changes | df | Mean square | | | | |
|  |  | Glycyrrhizic acid | Glabridin | Total phenolic content | Total flavonoid content | Total antioxidant (IC_50_) |
| Drought stress | 4 | 485.3^**^ | 0.027831^**^ | 82.57^**^ | 269.15^**^ | 1516.6^**^ |
| Seaweed extract | 2 | 60.6^**^ | 0.001202^**^ | 8.21^**^ | 9.18^**^ | 119.3^**^ |
| Drought stress × Seaweed extract | 8 | 3.2^**^ | 0.000133^**^ | 0.68^**^ | 2.54^**^ | 7.9^**^ |
| Residuals | 30 | 0.6 | 0.000093 | 0.4 | 0.92 | 3.2 |
| ^ns^ non-significant, **significant at P < 0.01 | | | | | | |

| **Table S2**. The analysis of variance (ANOVA) for biochemical traits of *Glycyrrhiza glabra* affected by drought stress and seaweed extract | | | | | | |
| --- | --- | --- | --- | --- | --- | --- |
| Sources of changes | df | Mean square | | | | |
|  |  | Proline | Ascorbate peroxidase (APX) | Catalase (CAT) | Superoxide dismutase (SOD) | Peroxidase (POD) |
| Drought stress | 4 | 1229.6^**^ | 475.2^**^ | 1104.8^**^ | 0.06547^**^ | 568.9^**^ |
| Seaweed extract | 2 | 32.8^**^ | 38.7^**^ | 2.6^**^ | 0.00228^**^ | 22.2^**^ |
| Drought stress × Seaweed extract | 8 | 4.1^**^ | 1.5^**^ | 0.7^**^ | 0.00018^**^ | 3.2^**^ |
| Residuals | 30 | 1.8 | 1.0 | 1.0 | 0.00017 | 1.0 |
| ^ns^ non-significant, **significant at P < 0.01 | | | | | | |
